# From Messengers to Receptors in Psoriasis: The Role of IL-17RA in Disease and Treatment

**DOI:** 10.3390/ijms22136740

**Published:** 2021-06-23

**Authors:** Silvia Vidal, Lluís Puig, José-Manuel Carrascosa-Carrillo, Álvaro González-Cantero, José-Carlos Ruiz-Carrascosa, Antonio-Manuel Velasco-Pastor

**Affiliations:** 1 Institute of Research, Hospital de la Santa Creu i Sant Pau, 08041 Barcelona, Spain; LPuig@santpau.cat; 2 Hospital Germans Trias i Pujol, 08916 Badalona, Spain; jmcarrascosac@hotmail.com; 3 Department of Dermatology, Hospital Universitario Ramón y Cajal, M-607, km. 9, 100, 28034 Madrid, Spain; alvarogc261893@hotmail.com; 4 Facultad de Medicina, Universidad Francisco de Vitoria, Ctra. Pozuelo-Majadahonda KM 1.800, 28223 Pozuelo de Alarcón, Spain; 5 Hospital Clínico Universitario San Cecilio, 18016 Granada, Spain; ruizcarrascosa@movistar.es; 6 Hospital Arnau de Vilanova, 46015 Valencia, Spain; m.velascop@telefonica.net

**Keywords:** psoriasis, Th17, IL-17, IL-17R, monoclonal antibodies, secukinumab, ixekizumab, bimekizumab, brodalumab

## Abstract

The paradigm of psoriasis as a Th17-driven disease has evolved in the last years towards a much deeper knowledge of the complex pathways, mechanisms, cells, and messengers involved, highlighting the crucial role played by the IL-17 family of cytokines. All IL-17 isoforms signal through IL-17R. Five subunits of IL-17R have been described to date, which couple to form a homo- or hetero-receptor complex. Characteristically, IL-17RA is a common subunit in all hetero-receptors. IL-17RA has unique structural—containing a SEFIR/TILL domain—and functional—requiring ACT-1 for signaling—properties, enabling Th17 cells to act as a bridge between innate and adaptive immune cells. In psoriasis, IL-17RA plays a key role in pathogenesis based on: (a) IL-17A, IL-17F, and other IL-17 isoforms are involved in disease development; and (b) IL-17RA is essential for signaling of all IL-17 cytokines but IL-17D, whose receptor has not been identified to date. This article reviews current evidence on the biology and role of the IL-17 family of cytokines and receptors, with focus on IL-17RA, in psoriasis and some related comorbidities, and puts them in context with current and upcoming treatments.

## 1. Psoriasis Pathogenesis in Context

Psoriasis is a chronic immune-mediated inflammatory skin disease whose prevalence in North America and Europe ranges from 1.50% to 1.92% [1]. The disease is histologically and clinically well-characterized; plaque psoriasis accounts for 80–90% of all manifestations [2,3]. This notwithstanding, knowledge on the pathogenesis of psoriasis has substantially evolved in the last two decades and is still improving.

Classical hypothesis of primary keratinocyte dysregulation [4] was replaced in the 80s by the immunologic hypothesis. Over the next two decades, relevant works (a) first identified T-cells accumulation in psoriatic lesions [5,6,7], (b) observed the improvement of skin lesions and reduction in inflammatory infiltrates by immunosuppressants cyclosporine A [8,9] and tacrolimus [10], and (c) demonstrated that blocking activated lymphocyte—not keratinocyte—proliferation improved disease manifestations [11]. The first description by Mosmann and colleagues [12] of two different subpopulations of murine CD4+ T helper cells (Th1 and Th2), and later demonstration of markedly increased expression of IL-12 (main cytokine involved in Th1 selection) in psoriatic plaques [13], underpinned the Th1-mediated pathogenic pathway of psoriasis. The immunologic basis for pathogenesis has led to a new field for psoriasis treatment research and paved the way for development of targeted monoclonal antibodies (´biologics´), starting with anti-TNF- α (a Th1 effector cytokine) antibodies as a successful therapeutic approach [14,15,16].

The Th1 pathogenic paradigm prevailed until cytokine IL-23 was discovered in 2000. Oppman and colleagues [17] described a novel protein p19 engaging protein p40 to form IL-23. Their work evidenced that p40 was a subunit shared by both IL-12 and IL-23, which differed in the second subunit. Some years later, a new subset of IL-23-driven CD4+ T cells, termed Th17, was first described [18]. IL-17A was considered the main effector cytokine of Th17 cells that, apart from their role against extracellular bacteria and fungi [19], were found to be involved in development and maintenance of autoimmune and chronic inflammatory diseases [20,21]. Interestingly, contemporary studies showed that early increase in IL-12 expression in psoriasis lesions was at the expense of the p40 subunit, whereas expression of p35, the other subunit, remained unaltered [22,23,24]. Thus, the Th1 pathogenic paradigm of psoriasis began to shift towards a Th17-driven disease, considering IL-23 and IL-17A as critical Th17 upstream and downstream cytokines, respectively. 

To date, growing knowledge has evidenced much more complexity within this pathway, with findings of paramount importance in psoriasis. T lymphocytes, as well as cells of the innate immune system and resident skin cells, can secrete IL-17. This implies a complex crosstalk between innate and adaptive immunity elements in the development of chronic inflammation [25]. Additionally, not only IL-17A, but five more IL-17 isoforms (IL-17B to F), are variably involved in the dysregulated inflammatory psoriatic response [26], meaning additional targets with potential implications for treatment. 

In light of this, IL-17 receptors (IL-17Rs) have attracted attention as therapeutic targets beyond IL-17 cytokines themselves. As explained below, IL-17 receptor family is structurally and functionally unique among interleukin receptors. Similar to IL-17 cytokines, receptors are dimeric proteins generally sharing one subunit (i.e., IL-17RA) and differing in the other (i.e., IL-17RB, IL-17RC, or IL-17RE). Interestingly, IL-17RA is a component of most dimeric IL-17R complexes [27]. Considering that almost all IL-17 cytokines (notably IL-17A, IL-17F, IL-17C, and IL-17E) have been shown to play some role in psoriasis pathogenesis, an approach based on receptor blockade, rather than cytokine inhibition, provides a consistent and valuable treatment strategy [27].

## 2. The IL-17 Pathogenic Route in Psoriasis

### 2.1. IL-17 Family Overview

IL-17 cytokines (IL-17s) are a family of dimeric proteins ranging from 34 to 52 kDa, of which six members have been described to date, termed consecutively from A to F [28]. 

IL17A was the founding member of the family, first identified and cloned from a murine cytotoxic T lymphocyte hybridoma cDNA library. It features an amino acid sequence unusual to other known cytokines, so that it was not considered initially a cytokine, and was named ´cytotoxic T lymphocyte-associated antigen 8′ (CTLA8) [29]. Subsequent sequence screenings searching for homologues led to identification of five more subtypes [30]. Homology related to IL-17A varies between the remaining subtypes, from the highest degree of sequence conservation (55%), found in IL-17F, to the lowest (16%), observed in IL-17E [28].

All IL-17s are secreted as homodimers, excepting IL-17A and IL-17F, which can also dimerize as a heterodimer IL-17A/F. Crystal structure studies have been performed on free IL-17F [31] and IL-17A [32]. Overall, the structure of both subtypes represents a cystine knot fold. Each monomer is structured as two anti-parallel β-sheets containing β-strands β1-β2 and β3-β4, two disulfide bridges connecting β2 and β4 in the C-terminal domain, and two serine residues, which are located where a third disulfide bridge used to be in the cysteine knot family of proteins. The N-terminal region contains the β0-strand, which is critical for maintaining a stable molecule. Monomers dimerize in a parallel way, similar to other cysteine knot proteins [31,32].

**Figure 1:**
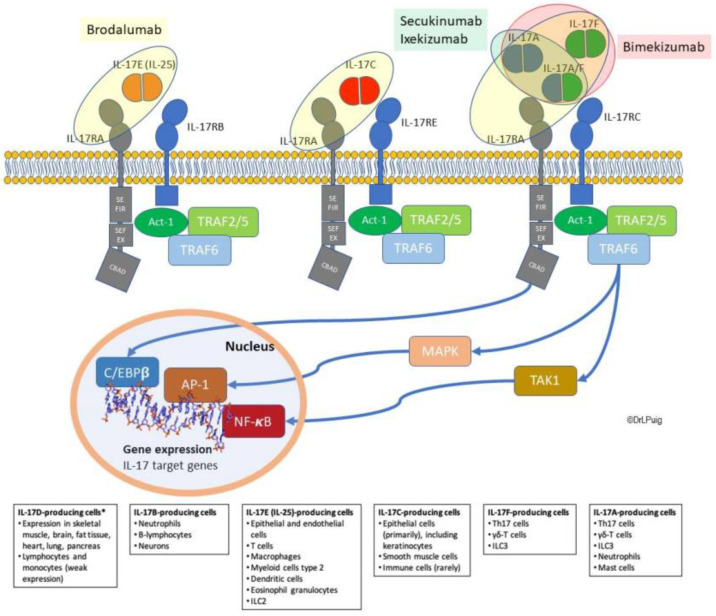
IL-17 family and treatments acting on the IL-17 pathway. Illustration of the different IL-17 ligands and receptors, and scheme of the intracellular signaling cascade (using an example of the activation of the IL-17A receptor). * IL-17D is not represented as its receptor remains unidentified; neither is IL-17B for the sake of simplicity. SEFIR: SEF/IL-17 receptor; SEFEX: SEFIR extension; CBAD: C/EBPß activation domain; and TRAF: TNF-receptor associated factor. Circles mean the blocking target of each therapy: those within yellow circles blocked by Brodalumab, those within green circle blocked by Secukinumab and Ixekizumab and those within pink circle blocked by Bimekizumab. This illustration has been elaborated from information available in references [26,27,30,36].

IL-17 cytokines play an important role in organ-specific immune response in a variety of physiologic functions and disorders, including host defense, inflammation, and autoimmunity [30]. To exert their action, IL-17A and IL-17F bind the heterodimeric IL-17 receptor (IL-17R) formed by subunits A and C (IL-17RA/RC); IL-17C signals through receptor IL-17RA/RE, whereas IL-17E does through IL-17RA/RB [33,34], and IL-17B has recently been found to bind the IL-17RA/IL-17RB complex [35]. To date, the receptor for IL-17D has not been elucidated (Table 1). The IL-17 family members, along with their receptors and targets for IL-17-targeted psoriasis treatments, are represented in Figure 1.

### 2.2. IL-17 Isoforms and Their Roles in Psoriasis

#### 2.2.1. IL-17A

IL-17A is a homodimeric glycoprotein of 155 amino acid length, encoded by a gene located at 6p12 [28] (Table 1). Numerous cells from both the adaptive and innate immune systems express IL-17A. It was primary identified as a signature cytokine of Th17 cells when this subset of CD4+ T lymphocytes was discovered. We know now that, once Th17 cells have been differentiated, amplified, and stabilized by the stimulus of polarizing upstream cytokines (mainly IL-1b, IL-6, TGF- β, IL-21, and IL-23) on RORC and IL-23R expression, activated Th17 cells secrete IL-17A and IL-17F, along with IL-21 and IL-22, as key effector cytokines. Unlike other T cells, Th17 differentiation is independent of its effector cytokine. Interestingly, IL-17A can also be secreted by Treg cells under stimulus of IL-23; this subset of T cells is essential for immune homeostasis and exhibits high plasticity. Other lymphocytes, including CD8+ and innate tissue-resident cells such as γδ-T, invariant NKT, NKT, and ILC3, are also primary sources of IL-17A. Among non-T cells, neutrophils, macrophages, and mast cells have also been reported to produce IL-17A under certain circumstances [34,37,38]. 

IL-17A plays a pivotal role in the pathophysiology of psoriasis, as it has prominent effects on epithelial cells, including keratinocytes. The activation of dermal DCs by several putative mechanisms triggers IL-23 secretion and recruitment of Th17 cells from lymphoid organs to the skin. These cells, along with tissue-resident lymphocytes activated by IL-23/IL23R binding as well, produce IL-17A and IL-17F to recruit neutrophils. Simultaneously, IL-17A induces keratinocytes to express pro-inflammatory cytokines, antimicrobial peptides, and chemokines that may directly recruit additional Th17, DCs, and neutrophils, thereby establishing a feed-forward inflammatory loop in lesional skin [33,37,39]. 

IL-17A is also involved in angiogenesis, another hallmark of psoriasis, as it is a known pro-angiogenic factor able to stimulate migration of endothelial cells as well as expression of VEGF. IL-17A may act synergistically with TNF- α to induce expression of IL-8; the latter two cytokines also have pro-angiogenic activity [40]. Heterodimeric IL-17A/IL-17F can also upregulate the expression of IL-8 and other pro-angiogenic factors, as well as induce migration of endothelial cells and even promote capillary-like endothelial tube formation [41].

#### 2.2.2. IL-17F

IL-17F is a homodimeric glycoprotein of 153 amino acid length, encoded by a gene located at 6p12 [28] (Table 1). As IL-17F and IL-17A genes are co-regulated and found at the same locus, both isoforms are frequently co-expressed by Th17 cells, γδ T cells, and ILC3, either as homodimers or heterodimers [26]. IL-17F binds to the same receptor as IL-17A; both share similar pro-inflammatory functions, and frequently act synergistically [33]. Nonetheless, IL-17F and IL-17A differ in other aspects. Different concentrations of their receptor subunits have been reported in myeloid versus non-myeloid cells, pointing towards different responses to IL-17A and IL-17F in both cell types [42]. The cytokine profile elicited by IL-17F has been also found to differ from IL-17A in experimental models of inflammatory arthritis [43]. The potency of IL-6 and IL-8 responses induced by IL-17F is generally weaker than that induced by IL-17A. However, both cytokines can act synergistically when co-present, inasmuch as their dual blockade results in a more marked decrease in IL-6 and IL-8 secretion than IL-17A blockade alone. Of interest, a IL-17F blockade alone had no effect on cytokines production [43]. 

In psoriatic lesions, expression of both IL-17F mRNA and protein is increased compared to non-lesional skin [44,45]. Furthermore, circulating concentrations of IL-17F are also higher in psoriatic patients than in healthy volunteers [45]. When IL-17F was studied in mice models of psoriasis, results showed that IL-17F-producing cells were more abundant than those producing IL-17A and IL-22. The importance of IL-17F in psoriasis development became more evident in mice lacking *Il17f* gene, which exhibited a more pronounced decrease in acanthosis than mice lacking *Il17a* [46].

#### 2.2.3. IL-17C

IL-17C is a homodimeric glycoprotein of 197 amino acid length, encoded by a gene located at 16q24 [28] (Table 1). Unlike prototypical IL-17A, epithelial cells, including keratinocytes, appear to be the main source for IL-17C [44,47], thus acting as a T cell-independent epithelial-derived stimulant of immune reactions. Other non-immune cells such as smooth muscle cells [48] also produce IL-17C. Importantly, it has been demonstrated that not only Th17 cells, but also cells of epithelial origin, express the IL-17RA/IL-17-RE complex—specific to the IL-17C receptor—on their surface [47,49]. This allows for an autocrine stimulation loop used by IL-17C to induce innate immune pathways in epithelial cells, with expression of cytokines and chemokines, inflammatory mediators, and antimicrobial peptides. Despite IL-17C similarities with IL-17A-mediated responses, there is no overlapping of functions in host defense mechanisms [47], where IL-17C stimulates highly inflammatory Th17 cells [49].

In psoriasis patients, *IL17C* mRNA is upregulated and expression of IL-17C increased compared to non-lesional or healthy skin; indeed, keratinocytes produce IL-17C in much higher amounts than IL-17A [47,50,51]. Interestingly, it has been observed that IL-17C stimulus induces endothelial cells to produce TNF- α ; in turn, the gene expression pattern induced by IL-17C/TNFα on keratinocytes is similar to that induced by IL-17A/TNFα [47,50]. Results from a recent study with an IL-23-induced mouse model of psoriasis have shown that functional neutralization of IL-17C using a specifically generated anti-IL-17C antibody resulted in a) reduced IL-23-induced skin inflammation and b) reduced gene expression of the aforementioned cytokine pattern in the skin, including IL-17A, IL-22, and IL-1β, among others [51]. 

Overall, the results suggest that bidirectional relationships between IL-17C and IL-17A would favor an inflammation loop in psoriatic skin, which may be controlled by inhibiting IL-17C.

#### 2.2.4. IL-17E

IL-17E, also named IL-25, is a homodimeric glycoprotein of 161 amino acid length, encoded by a gene located at 14q11.2 [28] (Table 1). IL-25 is secreted by a variety of cells, including epithelial cells, endothelial cells and several immune cells, such as T cells, macrophages, myeloid cells, DC, eosinophils, and ILC2 cells [26]. 

Although IL-25 is considered closer to Th2 immunity [30,52], it has been found to be strongly expressed in keratinocytes in psoriasis, playing a critical role in its development. Additionally, although an early study to characterize expression of IL-17 family members found undetectable IL17E mRNA in lesional skin [44], more recent studies have yielded opposite results. Xu et al. [53] compared biopsy specimens of psoriatic and normal human skin and observed a significant increase in both IL-25 mRNA and protein in lesional skin. Keratinocytes appear to be the main source of IL-25 in psoriasis, and its expression is tightly regulated by IL-17A. Results from animal models by the same author showed that IL-17A induced expression of *Il25* in keratinocytes, whereas its deficiency markedly reduced IL-25 levels. Interestingly, the inverse effect, i.e., IL-25 induction of IL-17A, was not observed. Moreover, other results point to the requirement of IL-25 expression in psoriasis. Mice lacking gene *Il25* exhibit greatly reduced epidermal acanthosis and dermal thickness, and significantly decreased development of pustules compared to control mice. Similar findings were observed in experiments using transgenic mice with selective ablation of *Il25* in keratinocytes. Furthermore, psoriatic mice treated with an anti-IL-25 neutralizing antibody showed significantly decreased psoriasiform skin inflammation. Additionally, IL-25 stimulation significantly promoted the expansion and cell-cycle progression of keratinocytes, whereas IL-25 deficiency greatly impaired proliferation of keratinocytes and epidermal hyperplasia [53]. A study in psoriasis and control patients [54], had earlier found highly increased levels of both IL-25 mRNA and protein in keratinocytes of psoriatic lesions, compared to non-lesional or control samples; furthermore, these IL-25+ cells outnumbered IL-17A+ cells in papillary dermis. This study identified macrophages as the main cells expressing IL-25 in psoriatic skin, despite the fact that they take IL-25 up via endocytosis rather than synthetize it. In response to IL-17E, the mRNA expression of several cytokines and chemokines, including TNF- α , IL-6, IL-8, and CCL20, increases in M2 macrophages in a dose-dependent manner, eventually resulting in recruitment of neutrophils. Indeed, the number of dermal IL-17E+ cells positively correlated with the number of neutrophils [54]. A later study from the same group [55] has recently demonstrated that IL-25 is not able to promote neutrophil recruitment on its own, but through a mechanism dependent on macrophage derived IL-8, suggesting an important role of IL-25 in neutrophil recruitment in skin inflammation.

Together, these findings (a) evidence the prominent role of immune cells, rather than epidermal keratinocytes, in mediating the IL-25-induced recruitment of neutrophils, (b) point to the role of macrophages as an immediate source of IL-25, and (c) support the critical role of this cytokine in psoriasis inflammation, with important implications in regards to psoriasis treatment. In this respect, it has been suggested that blocking IL-25 or its receptor could be a future therapeutic approach for Th17-mediated skin conditions [52].

#### 2.2.5. IL-17B

IL-17B is a homodimeric glycoprotein of 180 amino acid length, encoded by a gene located at 5q32-34 [28] (Table 1). Unlike IL-17A, activated T cells have not been found to express *IL17B* mRNA; rather IL-17B is expressed in neutrophils, germinal center B cells, neurons and stromal cells, and gut epithelium [56]. 

In psoriasis, the role of IL-17B is understudied, and the scarce evidence available showed a decrease in *IL17B* mRNA expression in lesional skin of psoriasis patients [44]. Nevertheless, IL-17B has been demonstrated to play a role in joint inflammation. In patients with rheumatoid arthritis, synovial tissues predominantly expressed IL-17B, and neutrophils therein were found to contain significant amounts of this cytokine. IL-17B induced the expression of IL-6, which is chemotactic to Th17 cells. It was also observed that fibroblasts respond to IL-17B, expressing IL-17RB, and that such expression is up-regulated under TNF- α stimulus. These results suggest an autocrine role for IL-17B in the maintenance of chronic joint damage, by promoting the proliferation and survival of neutrophils initially recruited by IL-17A [57]. On the other hand, results from a recent in vitro study have shown that NKT cells, CD4+ CRTH2+ Th2 cells, and ILC2s are targets of IL-17B, thus activating Th2 pathways and exhibiting pro-inflammatory activity. Indeed, these results suggest that IL-17B, although less potent than IL-25, shares many functional properties with this cytokine [35].

#### 2.2.6. IL-17D

IL-17D is a homodimeric glycoprotein of 202 amino acid length, encoded by a gene located at 13q12.11 [28] (Table 1). IL-17D is variably expressed in multiple human tissues, albeit it is poorly expressed in activated CD4+ and CD8+ T cells, suggesting that it has no role in the induction of immune cell proliferation. This notwithstanding, IL-17D can stimulate and modulate the production of other cytokines such as IL-6, IL-8, and GM-CSF by endothelial cells [58]. 

The role of IL-17D, not only in psoriasis but also in inflammatory diseases, is largely unknown. Similar to IL-17B, studies addressing IL-17D in psoriasis are scarce, but there is limited evidence of decreased levels in psoriatic skin lesions [44]. In other inflammatory diseases, such as rheumatoid arthritis, IL-17D has been detected in nodules, but not in synovial tissues [58].

### 2.3. IL-17 Isoforms beyond Psoriasis

Patients suffering psoriasis are at increased risk of developing other comorbidities, among which cardio-metabolic diseases are attracting great interest in recent years. Cardiovascular (CV) diseases, metabolic syndrome, and diabetes are prevalent among individuals with psoriasis [59,60]. A recent study has shown that psoriasis patients experiencing a myocardial infarction (MI) present a higher frequency of CV risk factors than their counterparts without psoriasis. Moreover, it suggests that psoriasis may act as a precipitating factor for MI [61]. 

Currently, it is acknowledged that these disorders share a link with chronic inflammation based on the activation of myeloid DCs and endothelial cells, promoting differentiation of Th1 and Th17 cells and secretion of pro-inflammatory cytokines such as TNF- α , IFN- γ , IL-17A, and IL-22, via activation of NF- κ B and MAPK signaling [62].

Increasing evidence suggests that IL-17A may represent one of the main links between CV disease manifestations and psoriatic inflammation [63,64]. In psoriasis mice models, dermal overexpression of IL-17A induced systemic endothelial dysfunction, vascular oxidative stress, and arterial hypertension. Furthermore, most mice suffered a premature death in relation to structural changes induced by hypertension. By contrast, mice treated with TNF- α and IL-6 inhibitors exhibited attenuation of oxidative stress and partial improvement of endothelial function, apart from reduction in skin lesions [65]. A recent investigation on the keratinocyte-endothelial cell relationship in psoriasis has found that IL-17A secreted by Th17 cells strongly induces the expression of IL-36, a pro-inflammatory cytokine, the main source of which are keratinocytes; this has been postulated to establish an endothelium-skin crosstalk in psoriasis. IL-36 activates dermal endothelial cells, which express receptors for both IL-17 and IL-36, inducing cell proliferation through ERK, STAT3, and NF-κB signaling, as well as expression of TNF- α -induced adhesion molecules such as ICAM-1. Additionally, IL-17A was found to strongly synergize with IFN-γ and TNF-α in stimulating the release of the angiogenic factor VEGF-A by human keratinocytes. The role of VEGF in psoriasis-associated angiogenesis as a nexus with CV disease is a topic of increasing interest [66]. Interestingly, VEGF-A expression in skin biopsy specimens from psoriasis patients was reduced after IL-17A inhibition with secukinumab [67]. These results confirm earlier findings [65,68], highlighting the involvement of IL-17A in endothelial damage concomitant with psoriasis and the potential role for IL-17 inhibitors. This has been further supported by a recent clinical study with psoriasis patients, in which improvement in vascular and myocardial function has been observed after IL-17A inhibition [69].

IL-17A is by far the most studied IL-17 isoform in psoriasis CV comorbidities. Nonetheless, some studies have addressed the role of other isoforms. Results from animal models showed that aortic smooth muscle cells and vascular cells produce IL-17C, which is elevated in atherosclerosis. IL-17C plays a pro-atherogenic role by inducing the expression of various chemokines and cytokines—including pro-Th17 CCL20, IL-6, IL-23p40, and IL-1β—within the aorta and stimulating the recruitment to the aortic wall of IL-17A expressing Th17 and TCRγδ+ T cells. Both IL-17A and IL-17C eventually induced the recruitment of monocytes and neutrophils to the plaque, thus increasing the cellularity of atherosclerotic lesions [48]. Lastly, IL-17E has been observed to exert pleiotropic effects on endothelium and atherosclerosis, as well as oxidation and inflammation processes, so that blocking this isoform could partially modify the pathogenesis of these conditions.

On the side of metabolic psoriasis comorbidities, it is worth mentioning NAFLD, the most common liver disease worldwide, potentially progressing to steatohepatitis and eventually cirrhosis. NAFLD is the hepatic manifestation of metabolic syndrome, both conditions being pathogenically linked by peripheral insulin resistance and chronic inflammation [70,71,72]. Importantly, the primary cause of mortality in NAFLD patients is cardiovascular disease [71]. 

Patients with psoriasis present higher prevalence of NAFLD and increased CV risk, and NAFLD risk is associated with psoriasis severity [72,73,74]. The IL-17 axis and IL-17-producing T cells have been implicated in NAFLD pathogenesis. Apart from skin manifestations, the pro-inflammatory effect of over-produced IL-17 contributes to adipogenesis and glucose metabolism dysregulation, resulting in inflamed, dysfunctional visceral adipose tissue and NAFLD [71,72]. Rau et al. found that progression from fatty liver to steatohepatitis was marked by an increase in IL-17^+^ cells in hepatic tissue, and by an increased Th17/rTreg ratio in both peripheral blood and liver tissue, supporting a central pathogenic role for the balance between Th17 and rTreg cells [75]. As previously mentioned with regard to CV comorbidities, IL-17A is the primary IL-17 family member studied in relation to NAFLD pathogenesis, along with IL-17RA [76,77]. Interestingly, upregulation of IL-17RA has been found in hepatic stellate cells and Kupffer cells in liver injury [77].

### 2.4. IL-17R as Unique Receptors in Cytokine Signaling: Focus on IL-17RA

The IL-17 receptor (IL-17R) family comprises five receptor subunits, correlatively named IL-17RA to IL-17RE, which are not homologous to any known receptors [27]. The receptor through which IL-17A, IL-17F, and IL-17AF signal is the IL-17RA/IL-17RC heteroreceptor. Interestingly, IL-17RA is a common subunit shared by other IL-17 receptors. Thus, IL-17C binds to the IL-17RA/IL-17RE heteroreceptor, and IL-17E (IL-25) to IL-17RA/IL-17RB. This notwithstanding, it has been recently shown that IL-17A also activates the IL-17RA/IL-17RD complex [78], inasmuch as IL-17B ligates IL-17RA/IL-17RB [35]. Unexpectedly, a recent in vitro study has reported that IL-17F, as well as IL-17A and IL-17A/F, can also bind two IL-17RC chains [79].

Structurally, all IL-17R subunits are single transmembrane domain-containing proteins in which certain conserved structural motifs are found, including an extracellular fibronectin type-III domain and a cytoplasmic SEF/IL-17R (SEFIR) domain, which is critical to mediate ligand signaling. A region at the C-terminal side of the SEFIR domain in IL-17RA has marked sequence homology to BB´ loops found in prototypical Toll/IL-1R (TIR) domains of TLRs from the innate immune system, therefore this motif is referred to as a TIR-like loop (TILL). It is presumed that these unique signaling properties of IL-17 receptors enable Th17 cells to act as a bridge between innate and adaptive immune cells. Of note, although all IL-17Rs have a SEFIR motif, the TILL domain seems to be unique to IL-17RA, which may explain why it is needed to partner with multiple IL-17R subunits [27,80].

Upon ligand binding, IL-17RA firstly recruits and activates the proximal adaptor protein ACT1, making another difference with Toll-like receptors. ACT1, which exhibits E3 ubiquitin ligase activity, also contains a SEFIR domain comprising a CC´ loop peptide that has been found to be critical for SEFIR-SEFIR interaction between IL-17RA and ACT1 [81]. ACT1 also bridges the interaction between IL-17RA and TRAF6, a signal transducer in the NF- κ B pathway with E3 ubiquitin ligase activity. ACT1-dependent signaling cascade induces the activation of NF- κ B, ERK, p38 MAPK, and JNK pathways. Interestingly, when IL-17A activates IL-17RA/IL-17RD instead of IL-17RA/IL-17RC heterodimers, p38 MAPK and JNK are preferentially induced [78]. Moreover, despite the crucial role of ACT-1, some ACT1-independent signaling in response to IL-17Rs activation has also been described [82,83]. 

Additional transcription factors are involved in response to IL-17A, including C/EBP β and C/EBP δ . The induction of C/EBPδ expression requires both ACT1 and the SEFIR and TILL domains of IL-17RA, which suggests the involvement of NF-κB, while C/EBPβ expression additionally needs a CBAD only found in IL-17RA. It must be mentioned that isoform LIP of C/EBP β is one of the few known inhibitory mechanisms of IL-17 signaling, activated by phosphorylation mediated by SEFIR, TILL, and CBAD domains of IL-17RA [27,49].

A fundamental aspect of IL-17 signaling is synergism and additive responsiveness, either between IL-17A and other cytokines (e.g., TNF-α, IL- 23, IL- 1β, IL- 6, and TGF- β) or between different IL-17 isoforms (e.g., IL-17F, IL-17C, and IL-17E), as the magnitude of the inflammatory effect cannot be explained as a mere sum of its components [39,50,84,85]. Regulation of mRNA stability is critical for coordinating cytokine expression and cytokine signaling, in which AU-rich elements (AREs) and GU-rich elements (GREs) as decay elements mediating rapid post-transcriptional degradation play a pivotal role [86]. It was initially found that activation of MAPKs by IL-17RA inhibited destabilization of proteins binding to AREs, thus blocking mRNA degradation and significantly increasing the concentration of related cytokines and chemokines [27]. Surprisingly, recent results have shown that ACT1 can also directly bind to mRNA, promoting its stability and translation [87]. Enhancement of mRNA stability may be one of the mechanisms underlying IL-17 synergism. Another mechanism for synergy involves C/EBP, as observed in functional cooperation between IL-17A and TNF-α [39].

Apart from characteristic signaling by activation of the aforementioned NF-κB, MAPK, and C/EBP pathways, IL-17RA can also signal through JAK/STAT. Upon IL-17E binding, the IL-17RA/IL-17RB complex has been found to activate STAT3 in an IL-6-independent, ACT1-dependent manner, resulting in upregulation of genes related to cell cycle progression and keratinocyte proliferation [53].

Additionally, IL-17RA interactions with a different type of receptor, such as epidermal growth factor receptor (EGFR), have been described [39]. Apart from known KRT16-mediated interplay, a recent study showed that IL-17RA, both functionally via ACT1, and physically by surface proximity, interacts with and transactivates EGFR in keratinocytes [88], suggesting a role in IL-17A-mediated keratinocyte hyperproliferation.

Expression of IL-17RA is ubiquitous, whereas its surface expression is widely variable [47,89,90]. IL-17RA mRNA has been found in murine spleen, kidney, liver, lung, brain, heart, skeletal muscle, and testes tissues, as well as in a wide variety of cell lines [91], although fibroblasts and endothelial cells have been reported to be the main responsive cells to IL-17A signal [89]. The broad distribution of IL-17RA, along with the large number of IL-17A-secreting cells and their migratory potential, underpin the IL-17A capacity to act on multiple cellular systems [47]. This contrasts with the localized expression of other IL-17 receptor subunits, such as IL-17RE, which is restricted to epithelial cells, thus limiting the activity of its ligand IL-17C to this cell type [47]. 

The expression of IL-17RA is dynamically modulated by the stimuli received by cells. IL-17RA expressed by CD8+ T-cells is induced by IL-15 and IL-21, and inhibited by IL-2 [92]. In dermal fibroblasts, UV irradiation induced a decrease in IL-6, IL-8, and CXCL-1 production simultaneously, with a decrease in IL-17RA and IL-17RC expression [93]. On the other hand, high levels of receptor expression are required for an effective IL-17RA response, implying a functional significance for receptor expression. Finally, IL-17RA internalization of its ligand IL-17 after binding is another mechanism by which IL-17RA may self-regulate its expression and signaling [89].

## 3. Targeting IL-17 Pathway in Psoriasis Treatment

### 3.1. Treatments Acting by Ligand Inhibition

#### 3.1.1. Secukinumab

Secukinumab was the first IL-17-targeted therapy approved for psoriasis. Secukinumab is a recombinant, fully human monoclonal IgG_1_ κ antibody designed to target and neutralize the actions of IL-17A. It binds the IL-17A homodimer with high affinity (K_D_ ~ 60—370 pM) and potency (0.14–0.4 nM), thus inhibiting its interaction with the IL-17RA receptor subunit. Although secukinumab is highly selective for IL-17A, it exhibits low cross-reactivity with the IL-17AF heterodimer (IC_50_ 3.3 nM), and even lower with IL-17F (IC_50_ ∼ 2 μM). Considering that IL-17F’s affinity to its receptor is substantially higher than to secukinumab, it is presumed not to contribute to secukinumab activity in therapeutic doses. Secukinumab does not cross-react with the other IL-17 family members [94].

Gene expression patterns from psoriasis lesional skin were analyzed in the psoriasis proof-of-concept study [95] after a single secukinumab dose of 3 mg/kg i.v., using quantitative RT-PCR and microarrays. Significant down-regulation of IL-12B, IL-17A, IL-17F, IL-21, IL-22, IFN- γ , CCL20, and TNF- α (by RT-PCR) and IL-6, IL-8, KRT16, and DEFB4 (by microarray analysis) was observed [95]. Regarding IL-17F, additional results from a 3-study pooled analysis found no consistent changes over time in the median serum IL-17F concentrations after secukinumab treatment [94].

The clinical efficacy of IL-17A inhibition by secukinumab in psoriasis has been extensively studied in multiple clinical trials. Briefly, a phase III study of secukinumab (150 mg and 300 mg) versus placebo in patients with moderate-to-severe plaque psoriasis demonstrated a significantly higher proportion of patients with PASI 75 at week 12 in the secukinumab groups (71.6% (150 mg) and 81.6% (300 mg)) than in the placebo group (4.5%; *p* < 0.001). Additional phase III studies demonstrated superiority of secukinumab over etanercept (PASI 75 at week 12: 67.0% (150 mg) and 77.1% (300 mg) vs. 44.0%; *p* < 0.001) and ustekinumab (PASI 90 at week 16: 79.0% (300 mg) vs. 57.6%; *p* < 0.001). Other relevant endpoints, such as improvement in PASI and IGA mod 2011 score were also significantly better with secukinumab across studies. However, when compared to guselkumab, the efficacy of the latter was superior (PASI 90 at week 48: 70.0% (300 mg) vs. 84.0%; *p* < 0.001) [96,97].

Overall, the safety and tolerability profile of secukinumab is favorable. As observed with other IL-17A inhibitors, an adjusted higher rate of *Candida* infections, mostly mucosal or cutaneous candidiasis, have been reported, which is consistent with the important role of IL-17A against fungal infections [98]. Paradoxically, case series of cutaneous inflammatory eruptions have been described from secukinumab—and ixekizumab—real life use [99,100]. Although the mechanism is yet unclear, it has been hypothesized that a Th1/Th2 imbalance favoring Th2 pathway, induced by IL-17A inhibitors and/or a IL-22-mediated mechanism—as IL-17A inhibition do not decrease IL-22 levels—might underlie these inflammatory eruptions [99]. More recently, they have been proposed to be a consequence of the selective block of IL-17A, which might induce the overexpression of isoform IL-17C, which is mainly produced by epithelial cells and seems to be involved in the pathogenesis of atopic dermatitis [100].

#### 3.1.2. Ixekizumab

Ixekizumab is a humanized IgG_4_ monoclonal antibody and the second approved drug designed to target and neutralize the actions of IL-17A. It selectively binds to IL-17A with high affinity (K_D_ < 3 pM). Ixekizumab neither binds IL-17F nor any of the other members of the IL-17 cytokine family [101].

The effect of IL-17 neutralization on gene expression was analyzed in an exploratory phase I study [102]. Expression of cytokines from multiple T cell subsets (Il-17A, IL-17F, IL-22, IFN- γ , IL-23/p19, and IL12/IL23p40), chemokines and antimicrobial peptides from keratinocytes (LL37, β -defensin 2, S100A7, and S100A8), as well as markers of keratinocyte proliferation (Ki67 and KRT16) were found to decrease after treatment in lesional skin from psoriasis patients.

The clinical efficacy of ixekizumab in psoriasis has been demonstrated in multiple clinical trials. Briefly, a phase III study of ixekizumab (80 mg Q2W and 80 mg Q4W) versus placebo in patients with moderate-to-severe plaque psoriasis resulted in a significantly higher PASI 75 response rate at week 12 in the treatment groups (89.1% and 82.6%, respectively) than in the placebo group (3.9%; *p* < 0.001). The percentage of patients with sPGA score of 0 or 1 (sPGA 0/1) was also significantly higher in the treatment groups. Two clinical trials compared both dose regimens of ixekizumab with etanercept, showing superiority for ixekizumab (PASI 75 at week 12: 87.3–89.7% (Q2W) and 77.5–84.2% (Q4W) vs. 41.6–53.4%; *p* < 0.001). Results from sPGA 0/1 also showed ixekizumab superiority. Comparisons with ustekinumab were addressed in an additional phase III study, which also demonstrated a significantly higher PASI 90 response rate at week 12 with ixekizumab, than with the active comparator (72.8% vs. 42.9%; *p* < 0.001). PASI 75, PASI 100, and sPGA 0/1 were relevant endpoints with also significantly higher response rates for ixekizumab compared to ustekinumab [97,103].

Overall, ixekizumab has a favorable safety profile and is well tolerated. As found with secukinumab, higher adjusted rates of *Candida* infections were described in ixekizumab clinical trials [104], and ‘paradoxical’ cutaneous eruptions have been reported in real life use [99,100].

#### 3.1.3. Bimekizumab

Bimekizumab (UCB4940) is an IL-17 inhibitor currently under phase III development for psoriasis. Unlike secukinumab and ixekizumab, which are specific for IL-17A, bimekizumab is a divalent humanized monoclonal IgG_1_ antibody designed to bind a similar site on both IL-17A (K_D_ 3.2 pM) and IL-17F (K_D_ 23 pM), thus conveying dual inhibition [105,106]. 

Transcriptional analysis of fibroblasts and synoviocytes from patients with psoriatic arthritis has been performed in an exploratory study. Bimekizumab, compared to an IL-17A inhibitor, induced a more profound anti-inflammatory effect in a large panel of inflammation-linked genes, including CCL20, CCL7, IL-6, IL-1b, and several chemokine ligands (CXCL), among others. The functional significance of dual blockade was confirmed by investigating the chemotactic potential of neutrophils towards Th17-stimulated fibroblasts. Dual IL-17A/IL-17F inhibition with bimekizumab achieved significantly more inhibition of neutrophil migration than neutralization of IL-17A or IL-17F alone. Additional results from the same study showed that, in the presence of TNF, both IL-17A and IL-17F (yet the latter with less potency) triggered a pro-inflammatory response with significantly increased production of IL-8 and IL-6. When these cells were exposed to bimekizumab as well as IL-17A, IL-17F, and TNF inhibitors, dual neutralization induced significantly greater down-regulation of IL-8, IL-6, and MMP3 than an IL-17A blockade alone, while an IL-17F blockade alone had no significant effect, and TNF inhibition had only a modest impact [106]. 

Results on clinical efficacy in plaque psoriasis are available from a phase I study in mild-to-moderate patients [105], and a phase IIb study in moderate-to-severe patients [107]. In the phase I study, bimekizumab doses ranging from 40 to 640 mg were compared to placebo [105], while in the phase IIb bimekizumab doses ranged from 64 mg to 480 mg, some of them preceded by a loading dose [107]. In mild-to-moderate patients, a mean reduction of >65% at week 2 from baseline PASI scores was observed in the 480 mg and 640 mg treatment groups. The maximum PASI improvement observed in these two groups was ≥94%. Bimekizumab 160 mg also improved PASI, though in a lesser magnitude. Comparable improvement was found in LSS and PGA scores. Responses to treatment were maintained to week 12. In moderate-to-severe patients, the percentage achieving PASI 90 at week 12 were significantly higher across all bimekizumab groups (46.2–79.1%) than in placebo group (0%; *p* < 0.0001). Other relevant endpoints, such as PASI 75, PASI 100, and PGA, were also significantly improved in bimekizumab groups compared to placebo [107]. 

Results from a phase III clinical trial comparing bimekizumab with adalimumab in moderate-to-severe plaque psoriasis patients have shown that bimekizumab was superior to adalimumab in reducing symptoms and signs of plaque psoriasis, but had a higher frequency of oral candidiasis and diarrhea [108]. Two more phase III studies with an active comparator have shown that bimekizumab is more effective than ustekinumab [109] and secukinumab [110]. 

### 3.2. Treatments Acting by Receptor Blockade

#### Brodalumab

Brodalumab is the first drug targeting a receptor instead of a ligand of the IL-17 pathway. Brodalumab is a human monoclonal IgG_2_ antibody that specifically binds IL-17RA with high affinity (K_D_ 0.24 nM) and blocks downstream signaling of multiple IL-17 family isoforms, including IL-17A, IL-17F, IL-17A/F heterodimer, and IL-25 [111].

Molecular and histological analyses of skin biopsy specimens in studies of subjects with psoriasis were conducted as part of three different clinical trials. The first was a phase I study evaluating different brodalumab single doses (i.e., 140 mg SC, 350 mg SC, and 700 mg IV) versus placebo [112]. Molecular results after brodalumab administration evidenced rapid and significant improvements in lesional skin mRNA levels for a number of IL-17-modulated keratinocyte-derived factors, including β-defensin 4, cathelicidin, KRT16, CCL18, and CCL20. Moreover, the mRNA levels of IL-17A, IL-17C, and IL-17F were reduced to non-lesional levels over 6 weeks; those of IL-22 and both subunits of IL-23 were also reduced. Histologically, significant reductions in epidermal thickening, KRT16 levels, and Ki67-expressing cells were observed. 

KRT16 and CD3 skin biomarkers were also measured in a biopsy sub-study of a phase II clinical trial evaluating brodalumab (doses of 70 mg, 140 mg, 210 mg, and 280 mg) versus placebo [113]. KRT16 staining of the upper epidermis was reduced in a dose-dependent manner across the 140 to 280 mg groups, and dermal CD3 counts decreased significantly from baseline across the 70 to 210 mg brodalumab groups. Additionally, epidermal thickness decreased significantly from baseline across the 140 to 280 mg groups.

The phase III study AMAGINE-1 [114], which evaluated brodalumab at doses of 140 mg and 210 mg versus placebo, also included a biopsy sub-study. Gene expression of IL-17 pathway cytokines (IL-17A, IL-17C, IL-17F, p19, and p40) decreased significantly at week 12 in a dose-dependent manner. Expression of IL-17C and both IL-23 subunits (IL-23p19 and IL-12p40) were reduced to near non-lesional levels, whereas IL-17A and IL-17F remained above non-lesional levels. From week 12 to 52, expression of all biomarkers in patients not re-randomized to placebo at week 12, continued decreasing, so that they were at or below non-lesional levels at week 52. Likewise, histological analysis evidenced that epidermal thickening, keratinocyte proliferation (Ki67, KRT16), and T-cell infiltrates (CD3 and CD8) were significantly reduced in samples from brodalumab groups at week 12 and continued improving up to week 52. Interestingly, samples from patients initially assigned to placebo and subsequently re-randomized to brodalumab showed no changes in biomarkers at week 12, but significant improvement at week 52, similar to that found in not re-randomized patients [114]. A mechanistic sub-study carried out with a subset of patients from three studies comprising the AMAGINE program was consistent with earlier results, showing a significant and progressive decrease in cellular infiltrates (CD31, CD81, CD11c1, and CD1631), markers of keratinocyte proliferation (Ki671 and KRT16), and inflammatory cytokines (IL-17A/C/F, p19, and p40), reaching close to non-lesional levels. Moreover, results confirmed that brodalumab induces transcriptional changes improving IL-17–dependent gene expression compared to ustekinumab, and such improvement correlated with PASI [115].

The clinical efficacy of brodalumab was thoroughly assessed over its clinical program, including one phase III clinical trial versus placebo (AMAGINE-1) and two phase III clinical trials versus ustekinumab (AMAGINE-2 and AMAGINE-3) [97,116]. Results from AMAGINE-1 indicated that, at week 12, a significantly greater proportion of patients in brodalumab groups achieved PASI 75 (60.3% (140 mg) and 83.3% (240 mg)) compared to placebo (2.7%; *p* < 0.001). Co-primary endpoint sPGA 0/1 was also significantly higher with brodalumab. The superiority of brodalumab over ustekinumab was also demonstrated. The AMAGINE-2 study showed that PASI 75 response at week 12 was greater with brodalumab (67% (140 mg) and 86% (240 mg)) than placebo (8%; *p* < 0.001). Co-primary endpoint sPGA 0/1 response was also significantly better with brodalumab than placebo. The second co-primary endpoint was PASI 100 response at week 12 compared to ustekinumab. Results demonstrated superiority of brodalumab 240 mg over ustekinumab (44% vs. 22%; *p* < 0.001). AMAGINE-3 reported similar results on PASI 75 at week 12 (69% (140 mg) and 85% (240 mg)) vs. 6% (placebo); *p* < 0.001) and sPGA 0/1, and met the PASI 100 co-primary endpoint at both doses (27% (140 mg) and 37% (240 mg) vs. 19%; *p* = 0.008 and *p* < 0.001, respectively). On the other hand, the uniqueness of brodalumab mechanism of action might explain its early onset of action, the fastest among biologics for psoriasis (<2 weeks) [117]. Furthermore, a recent systematic review and meta-analysis on long-term evidence available for the efficacy of systemic biologic and non-biologic drugs in moderate-to-severe psoriasis has estimated brodalumab as the most effective treatment in terms of both PASI response and probability of complete clearance [118].

Similar to secukinumab and ixekizumab, increased rates of *Candida* infections, mostly mucosal and cutaneous, and mild to moderate in severity, were reported from clinical trials [97]. In contrast, the risk for invasive fungal infections with these medications remains virtually close to zero to date, as no cases of deep fungal infections with IL-17-targeted treatments have been reported, according to a recent revision of randomized clinical trials including IL-12 and/or IL-23-targeted drugs [119]. The broad-targeting action of brodalumab rose concerns on its safety, based on the presumtion that blocking IL-17 isoforms besides IL-17A would increase the risk of impairing their additional physiological activity. Nevertheless, evidence from clinical trials and meta-analyses demonstrates that the safety profile of brodalumab is similar to those of other anti-IL17 biologics as well as ustekinumab [120,121]. 

### 3.3. Potential Role of IL-17A/IL-17RA Blockade in Psoriasis Comorbid Conditions

The potential effect of IL-17-targeted treatments on comorbidities, sharing with psoriasis a pathogenic role for the IL-17 pathway, is a field of growing interest to the available and upcoming anti-IL-17 biologics, considering some promising results observed earlier with anti-TNF α treatments [122]. 

In this regard, the impact of secukinumab has been assessed in an exploratory study including moderate-to-severe psoriasis patients without clinical CV disease and healthy volunteers [123]. Results indicated a significant improvement in endothelial function (determined as increased flow-mediated dilation) after 52 weeks of treatment with secukinumab compared to placebo. However, no significant changes in other relevant CV parameters were found. In the same vein, effects of ixekizumab on metabolic and CV-related markers and parameters have been evaluated, as well in moderate-to-severe psoriasis patients participating in three clinical trials for up to 60 weeks [124]. Results did not evidence consistent improvements over the study period. 

Neutralization of IL-17 has shown promising results on NAFLD in mice, inducing a decrease in pro-inflammatory cytokine levels, attenuation of hepatic lipid accumulation, and improvement of liver function [125,126] by a mechanism of IL-17-related fatty acid metabolism suppression [126]. On the other hand, it has been found that IL-17RA^−/−^ mice on a high-fat diet experience weight gain and hepatic triglyceride accumulation, but are protected from glucose metabolism dysfunction and progression of simple steatosis to steatohepatitis [76]. These findings suggest a potential role of IL-17A/IL-17RA blockade in comorbid NAFLD, but no clinical evidence in humans has been provided to date.

## 4. Conclusions

Psoriasis is a challenging systemic inflammatory disease that appears more complex with increasing knowledge of its pathogenic mechanisms, currently based on the critical involvement of IL-17 cytokines. Not surprisingly, a wide range of pharmacologic therapies are available for psoriasis treatment, including several biologics targeting the IL-17 pathway. Within this class of drugs, secukinumab and ixekizumab bind to and antagonize IL-17A and IL-17A/F, whereas brodalumab was developed to target and block the IL-17 receptor. By this unique mechanism, brodalumab binds IL-17RA and inhibits signaling of IL-17 isoforms, in addition to IL-17A and IL-17A/F, providing high efficacy, fast onset of action, and high probability of complete psoriasis clearance. Furthermore, the apparent pleiotropic effects of IL-17 isoforms signaling in psoriasis comorbidities is an exciting field of research that warrants future study.

## Figures and Tables

**Table 1:** Overview of IL-17 subtypes with characteristic length, encoding genes, effect on other cells, and association with diseases.

IL-17 Subtype	Length	Chromosomal Location	Effect on Other Cells	Association with Diseases
IL-17A	155	6p12	Proinflammatory effect on epithelial cells Synergy with IL-17F	Psoriasis Atopic eczema Multiple sclerosis Rheumatoid arthritis Psoriatic arthritis Chronic inflammatory bowel diseases Inflammation with acute coronary syndrome
IL-17F	153	6p12	Proinflammatory effect on epithelial cells	Psoriasis
			Synergy with IL-17A	Atopic eczema
				Multiple sclerosis
				Rheumatoid arthritis
				Psoriatic arthritis
				Chronic inflammatory bowel diseases
IL-17C	197	16q24	Autocrine stimulation of epithelial cells	Psoriasis
			Proinflammatory effect on epithelial cells via the expression of cytokines, chemokines, and antimicrobial peptides Synergy with TNF	Atopic eczema Rheumatoid arthritis Chronic inflammatory bowel diseases
IL-17E (IL-25)	161	14q11.2	Induces the loss of cellular barrier function Modulation of proinflammatory cytokines (e.g., IL-8, CCL-5, and GM-CSF) Reduced IL-17E expression in chronic inflammatory intestinal diseases	Psoriasis Atopic eczema Allergic contact dermatitis Bronchial asthma Rheumatoid arthritis Chronic inflammatory bowel diseases
IL-17B	180	5q32-34	Increase in TNF-α production by fibroblasts	Rheumatoid arthritis
			Poor prognosis in breast and stomach cancer	
IL-17D	202	13q12.11	Modulation of cytokine production by endothelial cells	Rheumatoid arthritis
			Release of proinflammatory cytokines (e.g., IL-6, IL-8, GM-CSF)	

## Abbreviations

CBAD	C/EBPβ-activation domain
C/EBP	CCAAT/enhancer-binding protein
CTLA8	Cytotoxic T lymphocyte-associated antigen 8
CV	Cardiovascular
DC	Dendritic cells
DEFB4	β-defensin 4
EGFR	Epidermal growth factor receptor
IGA mod 2011	2011 modified Investigator’s Global Assessment
ILC3	Group 3 innate lymphoid cells
JNK	c-Jun N-terminal kinase
LSS	Lesion Severity Score
NAFLD	Non-alcoholic fatty liver disease
PASI	Psoriasis Area and Severity Index
PGA	Physician´s Global Assessment
RORC	RAR-related orphan receptor
SEFIR	SEF (similar expression to fibroblast growth factor genes)/IL-17 receptor
TIR	Toll/IL-1 receptor
TLR	Toll-like receptor
TNF	Tumor necrosis factor
TRAF6	TNFR-associated factor 6
VEGF	Vascular endothelial growth factor
