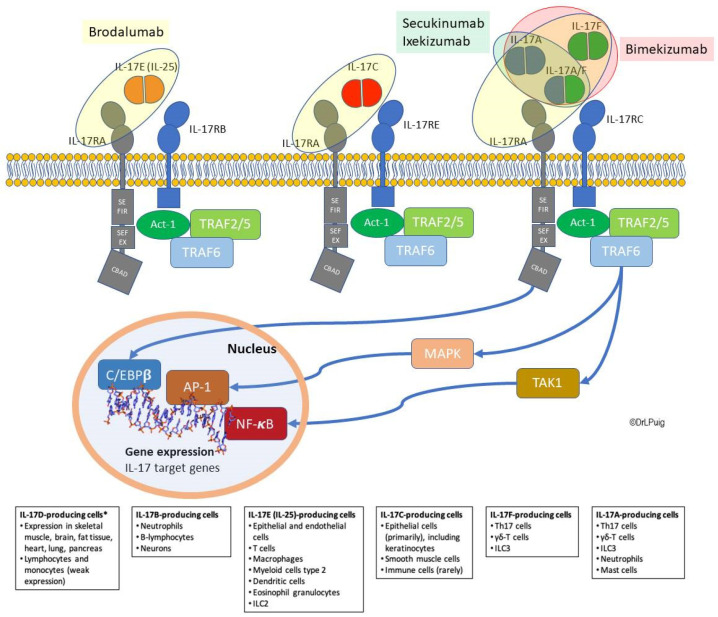# Correction: Vidal et al. From Messengers to Receptors in Psoriasis: The Role of IL-17RA in Disease and Treatment. *Int. J. Mol. Sci.* 2021, *22*, 6740

**DOI:** 10.3390/ijms23052649

**Published:** 2022-02-28

**Authors:** Silvia Vidal, Lluís Puig, José-Manuel Carrascosa-Carrillo, Álvaro González-Cantero, José-Carlos Ruiz-Carrascosa, Antonio-Manuel Velasco-Pastor

**Affiliations:** 1 Institute of Research, Hospital de la Santa Creu i Sant Pau, 08041 Barcelona, Spain; LPuig@santpau.cat; 2 Hospital Germans Trias i Pujol, 08916 Badalona, Spain; jmcarrascosac@hotmail.com; 3 Department of Dermatology, Hospital Universitario Ramón y Cajal, M-607, km. 9, 100, 28034 Madrid, Spain; alvarogc261893@hotmail.com; 4 Facultad de Medicina, Universidad Francisco de Vitoria, Ctra. Pozuelo-Majadahonda KM 1.800, 28223 Pozuelo de Alarcón, Spain; 5 Hospital Clínico Universitario San Cecilio, 18016 Granada, Spain; ruizcarrascosa@movistar.es; 6 Hospital Arnau de Vilanova, 46015 Valencia, Spain; m.velascop@telefonica.net

In the original publication [1], there was a mistake in Figure 1 as published. A new yellow circle has been added to clarify the action of brodalumab on IL-17A, A/F, and F. The three items named TRAF6 (colored in light green) have been substituted for the correct name: TRAF2/5. The corrected Figure 1 appears below. The authors apologize for any inconvenience caused and state that the scientific conclusions are unaffected. The original publication has also been updated.